# A Spatially Constrained Fibroblast–Myeloid Program Associates With Immune Exclusion and Poor Prognosis in Lung Adenocarcinoma

**DOI:** 10.1155/humu/8218916

**Published:** 2026-05-26

**Authors:** Yujun Zhou, Xinyang Ren, Rongxin Li, Sihan Chen, Bo Wei, Yi Shen, Xiang Wang, Yuzhen Xia, Siyu Cao, Pengpeng Sun, Nan Ding

**Affiliations:** ^1^ Institute of Pathogenic Biology, Hengyang Medical College, University of South China, Hunan Provincial Key Laboratory for Special Pathogens Prevention and Control, Hengyang, Hunan, China, usc.edu.cn; ^2^ Institute of Translational Medicine, School of Basic Medical Sciences, Hengyang Medical School, University of South China, Hengyang, Hunan, China, usc.edu.cn; ^3^ Department of Clinical Laboratory, Jining First People′s Hospital, Jining, Shandong, China

**Keywords:** cancer-associated fibroblasts, CCL3, CXCL8, immune exclusion, immunotherapy, lung adenocarcinoma, myeloid cells, periostin P, spatial transcriptomics

## Abstract

**Background:**

Although immune checkpoint blockade (ICB) therapy has improved clinical outcomes for some patients with lung adenocarcinoma (LUAD), only a subset of cases can achieve durable benefits, and primary resistance is often associated with an immune‐excluded tumor microenvironment (TME). Existing studies indicate that cancer‐associated fibroblasts (CAFs) are increasingly recognized as important participants in extracellular matrix (ECM) remodeling and stroma‐immune crosstalk. However, in LUAD, it is still unclear which CAFs‐related programs are involved in immune exclusion, particularly in relation to their spatial interactions with myeloid cell states.

**Methods:**

We integrated eight publicly available LUAD single‐cell RNA‐seq cohorts (164 samples; 471,501 cells) using Harmony and annotated major lineages and stromal subsets. CAFs were reclustered to resolve subtype heterogeneity, followed by pathway activity scoring, weighted gene coexpression network analysis (WGCNA), pseudotime trajectory inference (Slingshot), and regulon analysis. Bulk TCGA‐LUAD data were used for immune‐exclusion correlation and survival analyses. Spatial transcriptomics was applied for in situ validation, and ligand–receptor analysis together with NicheNet was used to prioritize CAFs‐derived signaling interactions and downstream targets.

**Results:**

The integrated atlas identified 10 major cell lineages and revealed tumor‐associated expansion of stromal and myeloid compartments. We further resolved six CAFs subtypes with distinct molecular and functional features. Tumor‐enriched CAFs subsets showed stronger activation of ECM remodeling, focal adhesion, TGF‐*β* signaling, hypoxia‐related pathways, and inflammatory programs. Bulk‐level analyses in TCGA‐LUAD demonstrated that CAFs‐related signatures were associated with a T‐cell exclusion index and increased expression of the immune checkpoint–related molecule CD276 (B7‐H3). Spatial transcriptomic mapping further showed that selected CAFs signatures were enriched in hypoxic, nonepithelial regions and colocalized with myeloid‐associated signals, supporting the presence of a spatially constrained fibro‐myeloid niche. Cell–cell communication analysis revealed extensive ligand–receptor interactions between CAFs subtypes and myeloid populations, whereas survival and immunotherapy cohort analyses showed that specific CAFs‐related programs were associated with worse clinical outcomes and less favorable treatment responses.

**Conclusions:**

Multicohort single‐cell integration and spatial validation define a CAFs‐centered, immune‐excluded niche in LUAD characterized by coordinated stromal and myeloid programs. These findings improve current understanding of CAFs heterogeneity and fibroblast–myeloid coupling in LUAD and provide a framework for future strategies aimed at targeting stromal barriers and remodeling the immune microenvironment to enhance ICB responsiveness.

## 1. Introduction

Lung adenocarcinoma (LUAD) remains one of the leading causes of cancer‐related death worldwide, while patients who can achieve durable clinical benefits from immune checkpoint blockade (ICB) therapy are still limited to a subset of individuals [1, 2]. An important biological reason for primary resistance is the immune‐excluded tumor microenvironment (TME), in which, despite the presence of effector T cells, they cannot effectively infiltrate tumor cell nests due to barriers from stroma, metabolism, and signaling pathways [3, 4]. In LUAD, this immune‐excluded structure is usually accompanied by hypoxia, extracellular matrix (ECM) remodeling, and enrichment of immunosuppressive myeloid programs, factors that collectively create a restrictive environment, thereby limiting antigen‐specific cytotoxicity and therapeutic responsiveness [5].

Among various stromal components, cancer‐associated fibroblasts (CAFs) are increasingly recognized as important organizers of immune exclusion. CAFs shape the TME through ECM deposition and stiffening, chemokine/cytokine secretion, and direct regulation of immune cell migration and function. In recent years, cross‐cancer single‐cell studies have revealed significant heterogeneity in CAFs (such as inflammatory CAFs, myofibroblastic CAFs, and antigen‐presenting CAFs), and the balance between different CAFs states can substantially affect immune infiltration, angiogenesis, and therapeutic sensitivity [6, 7]. However, the biological characteristics of CAFs are not solely determined intrinsically by the cells: Immune exclusion phenotypes often arise from mutual interactions between the stroma and the immune system, particularly the interactions between CAFs and macrophages. This interplay can further amplify fibrosis and immune suppression through feedback loops involving TGF‐*β*–related programs, ECM‐related ligands, and inflammatory mediators [8].

There is also increasing evidence indicating that CAFs do not act alone, but rather interact closely with myeloid cells to collectively construct a local microenvironment characterized by both fibrosis and immunosuppression [9]. Integrated single‐cell and spatial transcriptomic studies across multiple solid tumors suggest that fibroblast–myeloid cell interactions are often closely associated with spatial compartmentalization, ECM deposition, immune checkpoint–related immune dysfunction, and poor responses to immunotherapy. These observations support the notion that stromal programs and myeloid programs may work together to form anatomically confined local niches, thereby limiting effective antitumor immunity [10].

Against this background, there remains a need for systematic study of the heterogeneity of CAFs in LUAD. Although recent single‐cell studies have greatly deepened our understanding of stromal diversity in lung cancer, the functional states, regulatory programs, and spatial organization of tumor‐associated CAFs populations are still not fully elucidated, especially in the context of immune exclusion [11, 12]. Specifically, it remains unclear which CAFs subpopulations are most closely associated with stromal remodeling, hypoxia adaptation, and immune regulation, and how these stromal programs coordinate with myeloid cell states in the LUAD microenvironment [10, 13]. Moreover, recent studies in other solid tumors suggest that fibroblast–myeloid cell interactions may be involved in forming fibrotic and immune‐restricted local niches, which also implies the possible existence of similar cellular organizational structures in LUAD [14].

In this context, a comprehensive characterization of CAFs‐centered stromal remodeling in LUAD may provide important insight into the cellular basis of immune exclusion. Integrating large‐scale single‐cell transcriptomic data with trajectory analysis, regulatory network inference, cell–cell communication analysis, and spatial validation may help clarify how distinct CAFs states emerge, how they interact with myeloid populations, and whether these interactions are associated with anatomically constrained immune‐excluded regions. A better understanding of these fibroblast‐centered programs may also help identify candidate signaling pathways involved in fibrotic barrier formation and provide a rationale for developing therapeutic strategies to improve immunotherapy response in LUAD.

## 2. Materials and Methods

### 2.1. Data Collection and Cohorts

We integrated eight publicly available LUAD scRNA‐seq cohorts (GSE148071, GSE131907, GSE179994, GSE127465, GSE207422, GSE198099, GSE117570, and GSE223923), totaling 164 samples and 471,501 cells after quality control. For bulk‐level validation, TCGA‐LUAD RNA‐seq and associated clinical annotations were obtained from UCSC Xena. Spatial transcriptomics datasets used for validation were obtained from E‐MTAB‐13530 (https://www.omicsdi.org/dataset/biostudies-arrayexpress/E-MTAB-13530). All analyses were conducted on de‐identified, publicly available data; therefore, no additional ethics approval was required.

### 2.2. Single‐Cell Preprocessing, Integration, and Clustering

Raw count matrices from each cohort were processed in Seurat (v4.1.0). Cells with < 500 detected UMIs or > 15% mitochondrial transcripts were removed, and potential doublets were identified using the DoubletFinder function. Within each dataset, counts were normalized and log‐transformed; 2000 highly variable genes were selected and scaled. Datasets were integrated using Harmony (v 1.2.4) to mitigate batch effects. Principal component analysis (PCA) function was performed on the integrated object, and the top PCs were used to construct a shared nearest‐neighbor graph (FindNeighbors function) and perform graph‐based clustering (FindClusters function). Then uniform manifold approximation and projection (UMAP) was used for visualization.

### 2.3. Cell Type Annotation and Differential Expression

Major cell lineages and subclusters were annotated based on the expression of well‐established canonical markers. Specifically, T/NK cells were identified by CD3D, CD3E, and NKG7; epithelial cells by EPCAM and KRT19; B cells by CD79A and CD79B; fibroblasts by COL1A1, COL3A1, and ACTA2; myeloid cells by LYZ, FCGR3A, and CSF1R; endothelial cells by PECAM1, VWF, and PLVAP; mast cells by KIT and CPA3; ciliated cells by FOXJ1 and DNAH5; plasma cells by JCHAIN and MZB1; and proliferating cells by MKI67 and TOP2A. Cluster‐specific marker genes were then identified using Wilcoxon rank‐sum tests, followed by Benjamini–Hochberg correction for multiple testing, with commonly used cutoffs of min.pct = 0.25, logFC.threshold = 0.25, and only.pos = TRUE.

### 2.4. Tumor–Normal Composition Analysis

To quantify LUAD microenvironment remodeling, the fractions of major cell types were compared between tumor and adjacent normal tissues at the sample level. Differences were assessed using two‐sided Wilcoxon rank‐sum tests. Volcano plots and heat maps were generated from tumor‐versus‐normal DEG results and lineage‐specific signature genes.

### 2.5. CAFs Extraction and Subtype Definition

Fibroblast‐lineage cells were subsetted from the integrated single‐cell atlas and analyzed separately to further resolve CAFs heterogeneity. After reclustering, CAFs populations were annotated according to their representative marker genes, and subtype identities were recorded in the metadata as CAFs_subtype.

### 2.6. Functional State Scoring and Pathway Activity

To further evaluate the functional features of different CAFs subtypes, we collected curated gene signatures corresponding to several well‐recognized CAFs states, including iCAFs, vCAFs, aCAFs, pCAFs, myCAFs, and mCAFs. Enrichment of these signatures was calculated at the single‐cell level using AUCell. At the same time, ssGSEA was applied to score a series of biological programs related to angiogenesis, inflammatory response, PI3K–AKT signaling, TGF‐*β* signaling, proliferation, hypoxia, ECM organization, and myofibroblast differentiation. The overall functional profiles of different CAFs subtypes were then summarized and displayed by radar plots. To complement these analyses, PROGENy was also used, where appropriate, to infer pathway activity based on downstream target genes under settings recommended for single‐cell data.

### 2.7. Gene Set Enrichment Analysis

Subtype‐specific marker genes and pseudobulk differential expression results were subjected to Gene Ontology (GO) and Kyoto Encyclopedia of Genes and Genomes (KEGG) enrichment analyses using clusterProfiler. For GSEA, genes were ranked by signed effect size, and enrichment significance was assessed with FDR‐adjusted *p* values (< 0.05 considered significant).

### 2.8. Weighted Gene Coexpression Network Analysis (WGCNA/hdWGCNA)

To identify CAFs subtype‐associated transcriptional modules, we performed WGCNA on CAFs expression profiles using a scale‐free topology criterion to select the soft‐thresholding power. Modules were defined by hierarchical clustering of the topological overlap matrix and summarized by module eigengenes. Module‐subtype associations were quantified by correlating module eigengenes with CAFs subtype labels. Hub genes were prioritized by intramodular connectivity (e.g., kME) and visualized as coexpression networks for subtype‐enriched modules, including POSTN^+^ CAFs‐associated modules.

### 2.9. Trajectory Inference and Pseudotime Dynamics

CAFs trajectory analysis was performed using Slingshot on the UMAP embedding of the reclustered CAFs object. Specifically, variable features were used for PCA, followed by UMAP construction, and the resulting low‐dimensional representation was converted to a SingleCellExperiment object for trajectory inference. CAFs subtypes were used as cluster labels to infer lineage structure, and pseudotime values were then assigned back to the Seurat metadata for downstream visualization and analysis. For display purposes, pseudotime progression and trajectory‐related transcriptional states were visualized on the UMAP space. In some downstream analyses, cells were further grouped into branch‐associated bins along pseudotime to summarize dynamic expression patterns.

### 2.10. Transcriptional Regulatory Network Inference

Transcriptional regulatory analysis was performed at the CAFs subtype level to identify regulators associated with subtype‐specific transcriptional programs. Candidate transcription factors were prioritized based on their subtype‐enriched activity patterns and visualized using heat map‐based summaries in downstream analyses.

### 2.11. Immune Exclusion Metrics and Bulk Validation

For bulk‐level validation in TCGA‐LUAD, CAFs subtype signatures were quantified using ssGSEA/GSVA based on subtype marker genes. These subtype scores were then correlated with a T‐cell exclusion index using Pearson correlation analysis. In addition, samples were stratified according to subtype enrichment for downstream comparisons of genomic or immune‐related features, and group differences were evaluated using nonparametric tests where appropriate.

### 2.12. Spatial Transcriptomics Analysis

Spatial transcriptomic analyses were used to examine the spatial distribution of CAFs‐related programs and their relationship with local niche organization. Signature scores and selected marker genes were projected onto spatial coordinates to visualize compartmental localization and co‐occurrence patterns of stromal, epithelial, and immune‐associated features. Gene‐level spatial maps were further used to illustrate representative niche components and their spatial proximity.

### 2.13. Cell–Cell Communication and Ligand–Receptor Inference

Cell–cell communication analysis was performed to investigate interactions between CAFs subtypes and other cellular compartments, particularly myeloid populations. Ligand–receptor relationships and downstream signaling patterns were summarized in downstream visualizations to prioritize candidate intercellular signaling axes associated with CAFs‐centered niche remodeling.

### 2.14. Survival and Response Analyses

For clinical relevance analysis, subtype‐related scores derived from bulk transcriptomic data were used for downstream association analyses in TCGA‐LUAD. Samples were grouped according to CAFs subtype enrichment, and differences in outcome‐related or immune‐related features were subsequently evaluated.

### 2.15. Statistics and Reproducibility

Unless otherwise specified, statistical analyses were performed in R. Group comparisons were mainly conducted using nonparametric tests, whereas association analyses were evaluated using Pearson correlation where appropriate. Data processing and visualization were implemented with commonly used single‐cell analysis packages, including Seurat, Slingshot, GSVA, ggplot2, patchwork, and ComplexHeatmap.

### 2.16. T‐Cell Exclusion Index Analysis

To further assess the relationship between the POSTN^+^ CAFs program and immune suppression, a T‐cell exclusion index was calculated in the TCGA‐LUAD cohort based on a predefined exclusion‐related gene signature, including CD274, PDCD1, CTLA4, TIGIT, LAG3, HAVCR2, CD276, LGALS9, PDCD1LG2, TNFRSF8, IDO1, and ARG1. The enrichment score of this signature was estimated for each sample using ssGSEA, and the resulting score was used as a proxy for the degree of T‐cell exclusion. Pearson correlation analysis was subsequently performed to evaluate its association with the POSTN score.

### 2.17. Immunotherapy Cohort Analysis

To provide additional clinical validation in the immunotherapy setting, public immunotherapy cohorts were further analyzed. For each cohort, signature scores for programs were calculated and integrated with available clinical information. In IMvigor210, combined groups were defined according to POSTN^+^CAFs and CCL3^+^Mph scores, and survival differences were evaluated using Kaplan–Meier curves and log‐rank tests, followed by pairwise comparisons with BH adjustment when applicable. Pearson correlation analysis was also performed to examine the relationship between the two signatures. In GSE135222, patients were stratified according to signature scores using an optimal cutoff estimated by surv_cutpoint, or the median value when cut‐point estimation failed, and survival analysis was performed accordingly.

## 3. Results

### 3.1. Multicohort Integration Constructs a High‐Resolution Single‐Cell Atlas of LUAD and Reveals Tumor‐Associated Microenvironment Remodeling

**Figure 1:**
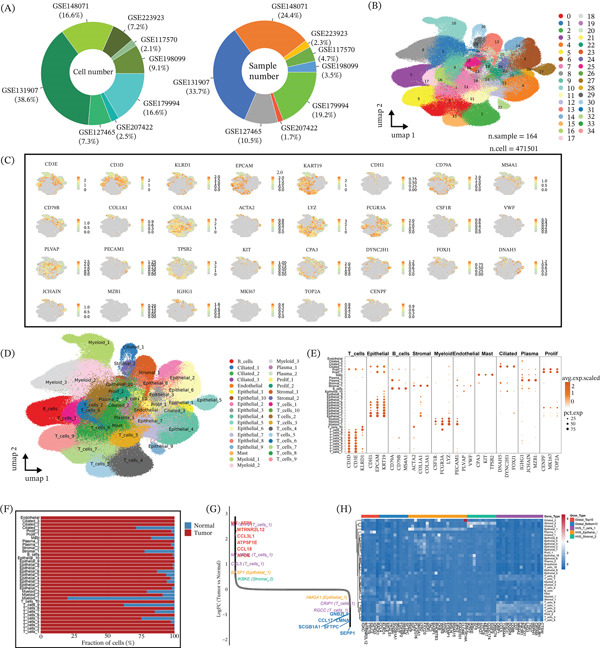
Integrated single‐cell transcriptomic landscape of lung adenocarcinoma reveals cellular heterogeneity and tumor microenvironment remodeling. (A) Pie charts showing the distribution of cell number (left) and sample number (right) across eight integrated scRNA‐seq datasets (GSE148071, GSE131907, GSE179994, GSE127465, GSE207422, GSE198099, GSE117570, and GSE223923). (B) UMAP visualization of integrated single‐cell data from 164 samples (471,501 cells) after batch correction, colored by 35 distinct cell clusters. (C) UMAP plots displaying the expression of canonical marker genes for major cell lineages, including T cells (CD3E, CD3D, and KLRD1), epithelial cells (EPCAM, KRT19, and CDH1), B cells (CD79A and CD79B), fibroblasts (COL1A1, COL3A1, and ACTA2), myeloid cells (LYZ, FCGR3A, and CSF1R), endothelial cells (VWF, PECAM1, and PLVAP), mast cells (KIT and CPA3), ciliated cells (DYNC2H1, FOXJ1, and DNAH5), plasma cells (JCHAIN, MZB1, and IGHG1), and proliferating cells (MKI67, TOP2A, and CENPF). (D) UMAP visualization of annotated cell types, including T cells, B cells, epithelial cells, stromal cells, myeloid cells, endothelial cells, mast cells, ciliated cells, plasma cells, and proliferating cells, with 35 transcriptionally distinct subsets. (E) Dot plot summarizing the expression of representative marker genes across all annotated cell types, with dot size representing the percentage of cells expressing the gene and color intensity indicating the average expression level. (F) Bar plot comparing the fraction of each major cell type in tumor versus adjacent normal tissues, showing the remodeling of cellular composition in the LUAD microenvironment. (G) Volcano plot of differentially expressed genes between tumor and normal tissues, highlighting key upregulated (e.g., MUC16 and MTRNR2L12) and downregulated genes. (H) Heat map displaying the expression profiles of signature genes across all annotated cell types, colored by cell lineage and sample type (tumor vs. normal).

We integrated single‐cell RNA‐seq data from eight independent LUAD cohorts (GSE148071, GSE131907, GSE179994, GSE127465, GSE207422, GSE198099, GSE117570, and GSE223923). Then, we further supplemented the integrated UMAP visualization for seven independent scRNA‐seq datasets to evaluate integration quality across cohorts. Cells were colored by dataset origin, and the integrated embedding showed substantial intermixing across batches with minimal dataset‐driven segregation, supporting effective batch correction and robust cross‐cohort integration (Figure S1A). After quality control and batch correction, a total of 471,501 cells from 164 samples were retained for downstream analysis, generating an integrated LUAD cell atlas (Figure 1A–B). Unsupervised clustering identified 35 transcriptionally distinct clusters, which could be grouped into 10 major cell lineages, including T cells, B cells, epithelial cells, fibroblasts, myeloid cells, endothelial cells, mast cells, ciliated cells, plasma cells, and proliferating cells (Figure 1B,D). Cell identities were assigned based on canonical marker genes, such as CD3E/CD3D for T cells, EPCAM/KRT19/CDH1 for epithelial cells, COL1A1/COL3A1/ACTA2 for fibroblasts, and LYZ/FCGR3A/CSF1R for myeloid cells (Figure 1C,E). We next compared the cellular composition between tumor and adjacent normal tissues at the sample level. Tumor samples showed higher proportions of stromal, myeloid, and proliferating cell populations than normal tissues (Figure 1F), suggesting substantial remodeling of the TME in LUAD. Consistent with this, differential expression analysis between tumor and normal tissues further identified a set of tumor‐associated transcriptional changes, with genes such as MUC16 and MTRNR2L12 among the representative upregulated features (Figure 1G). We also presented the expression patterns of marker genes across all annotated cell types, with cells colored by lineage and sample type (tumor vs. normal) (Figure 1H).

### 3.2. LUAD Harbors Six CAFs Subtypes With Distinct Molecular Identities and Functional Polarization, Including Tumor‐Enriched POSTN^+^ CAFs

**Figure 2:**
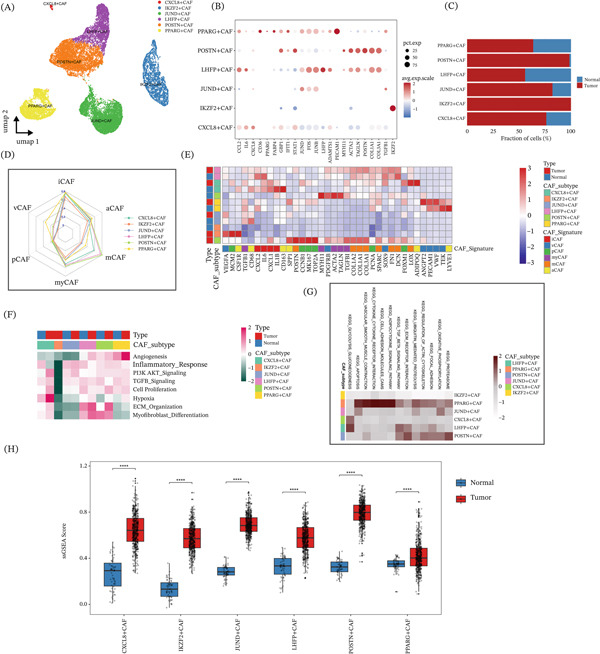
Transcriptional and functional heterogeneity of cancer‐associated fibroblasts (CAFs) in lung adenocarcinoma. (A) UMAP projection of all CAFs cells, colored by CAFs subtypes (CXCL8^+^CAFs, IKZF2^+^CAFs, JUND^+^CAFs, LHFP^+^CAFs, POSTN^+^CAFs, and PPARG^+^CAFs), illustrating the distinct clustering of each CAFs subset. (B) Dot plot showing the expression of key marker genes across different CAFs subtypes. The size of the dots represents the percentage of cells expressing the gene, and the color gradient represents the average expression level (low to high). (C) Stacked bar chart displaying the fraction of each CAFs subtype in normal and tumor samples, revealing the proportional distribution of CAFs subsets between normal and tumor microenvironments. (D) Radar plot illustrating the functional polarization of CAFs subtypes across six CAFs functional states (iCAFs, vCAFs, aCAFs, pCAFs, myCAFs, and mCAFs), with each line representing a distinct CAFs subtype. (E) Heat map of CAFs signature gene expression, with cells ordered by CAFs subtype and sample type (normal/tumor). The color gradient represents the normalized expression level (Z‐score) of each gene, and the top annotation bars indicate CAFs subtype and sample type. (F) Heat map of pathway activity scores (ssGSEA) for key CAFs‐related biological processes (angiogenesis, inflammatory response, PI3K‐AKT signaling, TGF*β* signaling, cell proliferation, hypoxia, ECM organization, and myofibroblast differentiation) across CAFs subtypes and sample types. The top annotation bars indicate sample type and CAFs subtype. (G) Heat map of transcription factor (TF) activity or gene set enrichment across CAFs subtypes and sample types, with cells ordered by CAFs subtype and sample type, and the color gradient representing the normalized activity score (Z‐score). (H) Box plot comparing the ssGSEA scores of CAFs signature pathways between normal (blue) and tumor (red) samples for each CAFs subtype. Statistical significance is indicated by asterisks ( ^∗∗∗∗^, *p* < 0.0001;  ^∗∗∗^, *p* < 0.001;  ^∗∗^, *p* < 0.01; and  ^∗^, *p* < 0.05).

To further resolve fibroblast heterogeneity, we reclustered stromal fibroblasts and identified six CAFs subtypes, namely CXCL8^+^ CAFs, IKZF2^+^ CAFs, JUND^+^ CAFs, LHFP^+^ CAFs, POSTN^+^ CAFs, and PPARG^+^ CAFs, based on their dominant marker genes (Figure 2A,B). Analysis of subtype composition showed that POSTN^+^ CAFs and IKZF2^+^ CAFs were preferentially enriched in tumor tissues compared with adjacent normal tissues (Figure 2C). We next examined the functional features of different CAFs subtypes using canonical CAFs‐related programs, including iCAFs, vCAFs, aCAFs, pCAFs, myCAFs, and mCAFs. Radar plot analysis suggested that POSTN^+^ and LHFP^+^ CAFs were more strongly associated with myCAFs‐like features, whereas the overall CAFs population displayed a more iCAFs‐like inflammatory profile (Figure 2D). Consistently, pathway activity analysis further showed that POSTN^+^ CAFs had the strongest activation of pathways related to ECM–receptor interaction, focal adhesion, PI3K–AKT signaling, and TGF‐*β* signaling. In addition, ssGSEA‐based pathway scoring revealed clear subtype‐specific differences in angiogenesis, inflammatory response, PI3K–AKT signaling, TGF‐*β* signaling, proliferation, hypoxia, ECM organization, and myofibroblast differentiation (Figure 2E,F). Among these subtypes, PPARG^+^ CAFs and POSTN^+^ CAFs showed particularly strong enrichment of angiogenesis‐related programs, which was also more evident in tumor tissues. Moreover, transcription factor activity was generally higher in PPARG^+^ CAFs and POSTN^+^ CAFs, whereas IKZF2^+^ CAFs appeared relatively inactive in ECM‐related and other biological programs, with overall low transcription factor activity as well (Figure 2G).

When tumor‐derived and normal‐derived CAFs were compared, tumor‐associated CAFs showed significantly higher pathway activity scores across subtypes (Figure 2H), supporting a globally activated and protumorigenic stromal state in LUAD. It is noteworthy that POSTN^+^ CAFs exhibit the highest activity in multiple key pathways, especially in the TGF‐*β* signaling pathway, ECM–receptor interaction, and focal adhesion, suggesting that this subpopulation may play an important role in coordinating these matrix‐related functions in the LUAD microenvironment.

### 3.3. WGCNA Defines Subtype‐Specific CAFs Coexpression Modules and Highlights POSTN^+^ CAFs‐Centered Regulatory Programs

**Figure 3:**
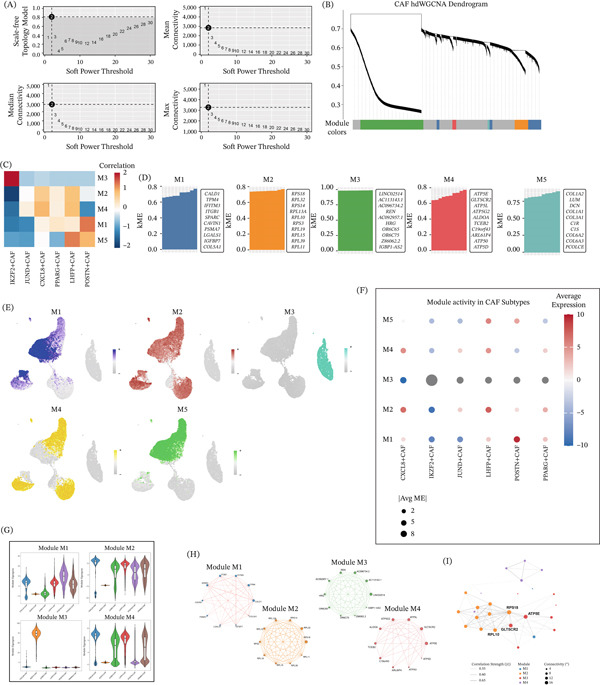
Weighted gene coexpression network analysis (WGCNA) identifies subtype‐specific functional modules and regulatory networks in cancer‐associated fibroblasts (CAFs). (A) Selection of soft‐thresholding power for WGCNA. Plots show scale‐free topology model fit (top left), mean connectivity (top right), median connectivity (bottom left), and max connectivity (bottom right) as functions of the soft power threshold. The selected soft power (black dot) ensures a scale‐free network structure. (B) Hierarchical clustering dendrogram of CAFs cells (hdWGCNA), with module colors assigned to distinct gene coexpression modules (M1–M5). (C) Heat map of correlation coefficients between WGCNA modules (M1–M5) and CAFs subtypes. Color gradient represents correlation strength and direction (blue: negative; red: positive). (D) Bar plots of eigengene connectivity (KME) for genes within each module (M1–M5). Top hub genes (highest KME) are annotated for each module. (E) UMAP visualization of CAFs cells colored by module eigengene (ME) expression for M1–M5, showing spatial distribution of module activity across the CAFs population. (F) Dot plot of module activity (measured by |Avg ME|) across CAFs subtypes. Dot size corresponds to |Avg ME|, and color gradient represents average gene expression within the module (blue: low; red: high). (G) Violin plots of module activity (M1–M4) stratified by CAFs subtype, showing module eigengene expression distribution across CAFs subsets. (H) Coexpression network visualization for modules M1 (pink), M2 (orange), M3 (green), and M4 (red). Nodes represent hub genes; edges represent significant coexpression (correlation strength > 0.8). (I) Coexpression network visualization for POSTN^+^ CAFs. Node size is proportional to gene connectivity (degree); edge thickness represents coexpression strength (correlation coefficient).

To further characterize transcriptional heterogeneity across CAFs, a soft‐thresholding power of 2 was selected to achieve a scale‐free topology (R^2^ = 0.9) (Figure 3A). Based on this parameter, five independent coexpression modules (M1–M5) with distinct gene expression patterns were identified (Figure 3B). Correlation analysis showed clear subtype preferences for these modules, with individual modules preferentially associated with specific CAFs subsets. Among them, POSTN^+^ CAFs showed the strongest correlation with M1, which was characterized by genes such as CALD1, TPM4, and ITGB1 (Figure 3C). Intramodular hub genes were further prioritized according to module membership (kME) (Figure 3D). In addition, to better interpret the biological significance of the five WGCNA modules (M1–M5), we performed GO and KEGG enrichment analyses for module genes. Bar plots were used to summarize the top enriched terms from biological process (BP), cellular component (CC), molecular function (MF), and KEGG pathways for each module. In these plots, the color gradient indicates enrichment significance, and bar length represents the gene ratio (Figure S1B–F). These analyses provided a more systematic functional annotation of module‐specific transcriptional programs and further supported the biological interpretation of distinct CAFs‐associated coexpression modules. Projection of module eigengene activity onto the UMAP revealed distinct distribution patterns across the CAFs population (Figure 3E). In addition, network visualization of representative modules highlighted densely connected hub genes within each module, providing a coexpression‐based framework for understanding subtype‐associated transcriptional programs in CAFs (Figure 3F,G). Based on the downstream analyses and the particular relevance of the POSTN‐high state, we further revised the presentation to show the coexpression networks of M1, M2, M3, and M4 in the main figure, whereas the remaining module networks are provided in Figure S1G. The identification of these core regulatory genes provides important insight into the molecular basis underlying the characteristics and functions of different CAFs subsets in the TME. Notably, ATP5E and GLTSCR2 emerged as highly connected hub nodes, suggesting that they may play important roles in maintaining network structure and stability, and further deepening our understanding of CAFs subtype specialization in LUAD (Figure 3H,I). Overall, this network‐based analysis revealed the complex transcriptional architecture underlying CAFs heterogeneity and provided a basis for subsequent investigation of pathway activity and regulatory mechanisms.

### 3.4. Pseudotime and Regulon Analyses Reveal CAFs Differentiation Trajectories and Nominate FOXS1 and IL24 as Subtype‐Specific Regulators

**Figure 4:**
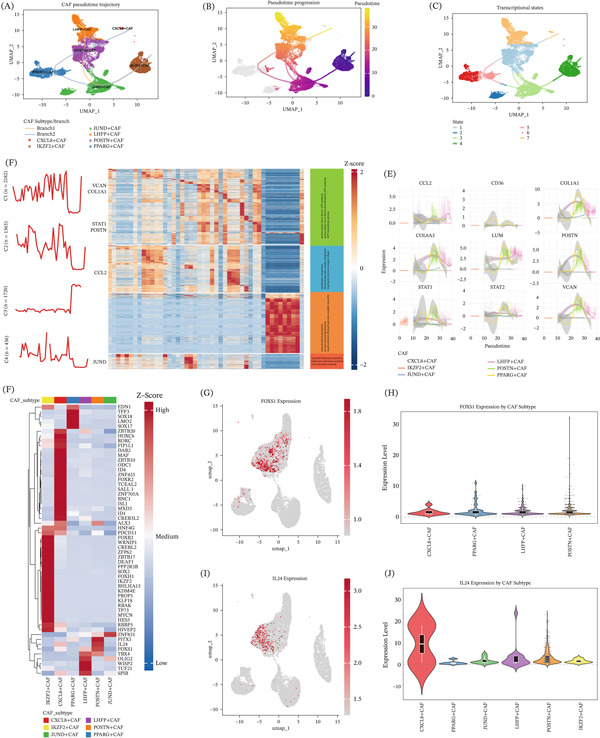
Pseudotime trajectory and transcriptional regulatory network analysis uncovers CAFs differentiation progression and core regulators in lung adenocarcinoma. (A) UMAP visualization of CAFs pseudotime trajectory inferred by Slingshot, colored by CAFs subtypes (CXCL8^+^CAFs, IKZF2^+^CAFs, JUND^+^CAFs, LHFP^+^CAFs, POSTN^+^CAFs, and PPARG^+^CAFs), with trajectory lines indicating the differentiation branches. (B) UMAP plot of CAFs pseudotime progression, with cell color gradient representing pseudotime values (low to high) to reflect continuous differentiation. (C) UMAP visualization of seven distinct transcriptional states identified by clustering along the pseudotime trajectory, colored by state. (D) Fine‐grained heat map of pseudotime‐related differentially expressed (DE) genes in CAFs, with genes clustered into four expression modules (C1–C4). Left: Average expression trends of each module along pseudotime, with key core genes (VCAN, COL1A1, STAT1, POSTN, CCL2, and JUND) labeled. Right: Functional annotations (GO biological processes) for each module. Columns represent pseudotime bins across two differentiation branches. (E) Expression trends of core CAFs functional genes along pseudotime, with each subplot showing the distribution (dots) and loess‐fitted trend line (smooth) of gene expression, colored by CAFs subtype. (F) Heat map of transcription factor (TF) regulon activities inferred by R‐SCENIC, with cells ordered by CAFs subtype to display distinct TF regulon usage patterns across CAFs subsets. Color gradient represents normalized activity (Z‐score). (G) UMAP feature plot of FOXS1 expression in CAFs, with color gradient representing expression level from low (gray) to high (red). (H) Violin plot of FOXS1 expression stratified by CAFs subtype, showing the expression distribution across different CAFs subsets. (I) UMAP feature plot of IL24 expression in CAFs, with color gradient representing expression level from low (gray) to high (red). (J) Violin plot of IL24 expression stratified by CAFs subtype, showing the expression distribution across different CAFs subsets.

Trajectory inference using Slingshot reconstructed the dynamic progression of CAFs and revealed two major differentiation branches (Figure 4A–B). Unsupervised state assignment along pseudotime further identified seven transcriptional states. The inferred lineage topology suggested a branched differentiation pattern, with IKZF2^+^ CAFs located near the root, JUND^+^ CAFs occupying an intermediate position, and LHFP^+^ and POSTN^+^ CAFs representing terminal states. Pseudotime ordering indicated a gradual transition from relatively quiescent fibroblast states toward activated phenotypes characterized by ECM production and immune‐modulatory features (Figure 4C). Pseudotime‐associated differentially expressed genes were grouped into four temporal expression modules (C1–C4), showing sequential activation of programs related to ECM/focal adhesion (e.g., VCAN and COL1A1), inflammatory and TGF‐*β*–associated signaling (e.g., STAT1 and POSTN), immune recruitment (e.g., CCL2), and stress/proliferation‐related responses (e.g., JUND) (Figure 4D,E). We also observed that FOXS1 and IL24 were strongly associated with POSTN^+^ CAFs (Figure 4F). UMAP visualization further showed that both genes were markedly enriched in the POSTN^+^ CAFs cluster compared with other fibroblast subtypes, although they were also expressed in additional subsets (Figure 4G,I). In particular, we also showed the expression patterns of these two transcription factors in other CAFs subtypes, and IL24 was also prominently expressed in CXCL8^+^ CAFs (Figure 4H,J). Together, these findings suggest a continuous differentiation spectrum from relatively quiescent fibroblasts to terminal CAFs states, and nominate FOXS1 and IL24 as candidate transcriptional regulators associated with stromal remodeling and immune‐excluded microenvironments.

### 3.5. POSTN^+^ and CXCL8^+^ CAFs Programs Associate With Immune Exclusion and Localize to Hypoxic, Nonepithelial Regions in Spatial Transcriptomics

**Figure 5:**
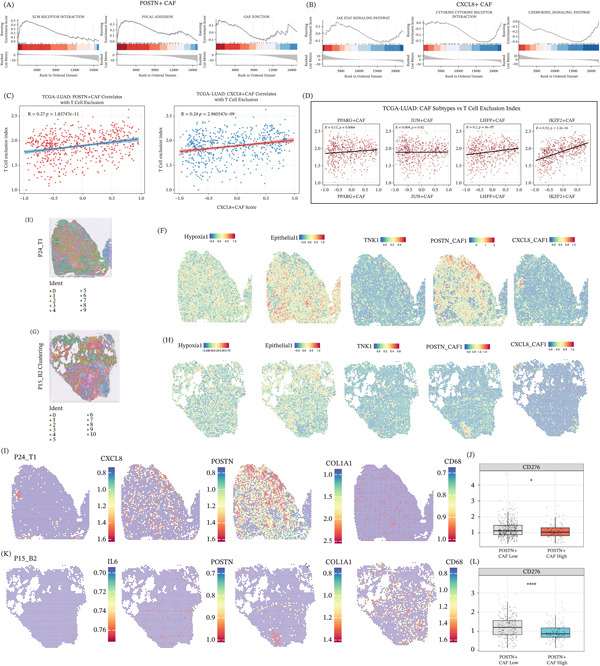
Functional enrichment and spatial characterization of CAFs subtypes in lung adenocarcinoma. (A) Gene set enrichment analysis (GSEA) of POSTN^+^ CAFs cells from scRNA‐seq data, showing significant enrichment of pathways including ECM–receptor interaction, focal adhesion, and gap junction. Normalized enrichment scores (NES) and *p* values are indicated. (B) GSEA of CXCL8^+^ CAFs cells from scRNA‐seq data, showing significant enrichment of pathways including JAK‐STAT signaling pathway, cytokine–cytokine receptor interaction, and chemokine signaling pathway. NES and *p* values are indicated. (C) Scatter plot showing the positive correlation between POSTN^+^ CAFs signature score and T‐cell exclusion index in the TCGA‐LUAD cohort. Pearson correlation coefficient (R) and *p* value are annotated. (D) Scatter plots showing the correlation between CAFs subtype signature scores (POSTN^+^ CAFs, CXCL8^+^ CAFs, PPARG^+^ CAFs, JUND^+^ CAFs, IKZF2^+^ CAFs, and LHFP^+^ CAFs) and T‐cell exclusion index in the TCGA‐LUAD cohort. Regression lines and *p* values are indicated for each subtype. (E) Spatial transcriptomics visualization of section P24_T1, colored by unsupervised clustering of cell states. (F) Spatial distribution of signature scores for hypoxia, epithelial, TNK1, POSTN^+^ CAFs, and CXCL8^+^ CAFs in section P24_T1, overlaid on tissue histology. Color gradient represents signature score intensity. (G) Spatial transcriptomics visualization of section P16_B2, colored by unsupervised clustering of cell states. (H) Spatial distribution of signature scores for hypoxia, epithelial, TNK1, POSTN^+^ CAFs, and CXCL8^+^ CAFs in section P16_B2, overlaid on tissue histology. Color gradient represents signature score intensity. (I) Spatial expression heat maps of CXCL8, POSTN, COL1A1, and CD68 in section P24_T1, overlaid on tissue histology. Color gradient represents gene expression level. (J) Box plot comparing CD276 (B7‐H3) expression levels between POSTN^+^ CAFs low and high groups in the TCGA‐LUAD cohort. Statistical significance is indicated by asterisks ( ^∗^, *p* < 0.05). (K) Spatial expression heatmaps of IL6, POSTN, COL1A1, and CD68 in section P15_B2, overlaid on tissue histology. Color gradient represents gene expression level. (L) Box plot comparing CD276 (B7‐H3) expression levels between POSTN^+^ CAFs low and high groups in an independent spatial transcriptomics cohort. Statistical significance is indicated by asterisks ( ^∗∗∗∗^, *p* < 0.0001).

GSEA indicated that POSTN^+^ CAFs were enriched for ECM–receptor interaction, focal adhesion, and gap junction pathways (Figure 5A), whereas CXCL8^+^ CAFs were enriched for JAK–STAT signaling, cytokine–cytokine receptor interaction, and chemokine signaling (Figure 5B). In TCGA‐LUAD, the POSTN^+^ CAFs signature score correlated positively with a T‐cell exclusion index (Figure 5C), and subtype‐level analyses showed that POSTN^+^ and CXCL8^+^ CAFs signatures were among the most strongly associated with immune exclusion (Figure 5D). Spatial transcriptomics validated these relationships in situ: POSTN^+^ and CXCL8^+^ CAFs signature scores localized predominantly to hypoxic, nonepithelial regions, whereas epithelial programs concentrated in tumor‐cell–rich compartments (Figure 5E–H). Spatial feature plots further showed the colocalization of POSTN/CXCL8, the CAFs marker COL1A1, and the CD68^+^ myeloid signal (Figure 5I,K), consistent with a spatially constrained fibro‐myeloid niche. Finally, in both the TCGA‐LUAD cohort (Figure 5J) and an independent spatial cohort (two‐sided Wilcoxon rank‐sum test), CD276 (B7‐H3) expression differed significantly between the POSTN^+^ CAFs‐high and POSTN^+^ CAFs‐low groups (Figure 5L), supporting differences in the enrichment of an immunosuppressive checkpoint‐associated microenvironment according to the abundance of POSTN^+^ CAFs.

### 3.6. Ligand–Receptor and NicheNet Analyses Uncover a CAFs–Myeloid Communication Network Dominated by POSTN^+^ CAFs‐Derived Ligands and CCL3^+^ Myeloid Programs

**Figure 6:**
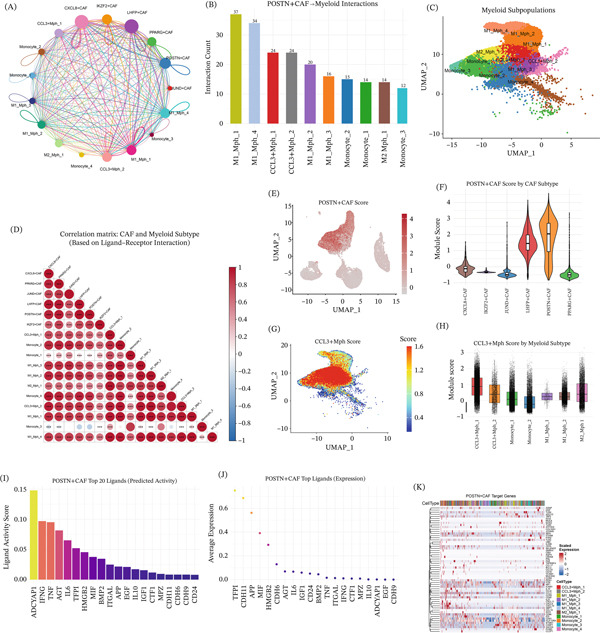
Intercellular communication analysis reveals ligand–receptor interactions between CAFs subtypes and myeloid cells in lung adenocarcinoma. (A) Circle plot visualization of intercellular communication networks in the integrated LUAD single‐cell atlas, illustrating significant ligand–receptor interactions between CAFs subtypes (POSTN^+^ CAFs, CXCL8^+^ CAFs, PPARG^+^ CAFs, JUND^+^ CAFs, IKZF2^+^ CAFs, and LHFP^+^ CAFs) and major myeloid subpopulations (Monocyte_1 to Monocyte_5). Node size corresponds to interaction strength, and edge color indicates the participating CAFs–myeloid cell pairs. (B) Bar chart quantifying the number of predicted ligand–receptor interactions between POSTN^+^ CAFs and distinct myeloid cell subtypes (Monocyte_1 to Monocyte_5). Error bars represent standard deviations. (C) UMAP projection of myeloid cell subpopulations in the integrated LUAD single‐cell atlas, colored by distinct myeloid subtypes (M1–M6). (D) Heat map of correlation coefficients between CAFs subtype signature scores and myeloid cell subtype abundances based on ligand–receptor interaction pairs. Color gradient represents correlation strength (red: positive; blue: negative). (E) UMAP feature plot of POSTN^+^ CAFs signature scores. Color gradient indicates signature score intensity. (F) Violin plots showing the distribution of POSTN^+^ CAFs signature scores across distinct CAFs subtype subpopulations. (G) UMAP feature plot of CCL3^+^ myeloid signature scores overlaid on myeloid cell UMAP coordinates. Color gradient indicates signature score intensity. (H) Violin plots showing the distribution of CCL3^+^ myeloid signature scores across distinct myeloid subpopulations (M1–M6). (I) Bar plot ranking the top 20 predicted ligands derived from CAFs subtypes according to interaction strength. (J) Lollipop plot visualizing the top predicted ligand–receptor pairs from CAFs subtypes, with dot size representing interaction strength and color indicating the corresponding CAFs subtype. (K) Heat map showing the expression patterns of predicted target genes downstream of POSTN^+^ CAFs‐associated ligands across CAFs subtypes. Color gradient represents normalized expression (Z‐score).

Intercellular communication analysis revealed extensive ligand–receptor interactions between CAFs subtypes and multiple myeloid subpopulations (Figure 6A). Among these interactions, POSTN^+^ CAFs showed the strongest communication with Monocyte_1 (Figure 6B). We then visualized the distribution of myeloid subpopulations on the UMAP (Figure 6C,D). The POSTN^+^ CAFs signature score was calculated and displayed within the CAFs compartment using a CAFs‐only Seurat object (Figure 6E,F). In parallel, the CCL3^+^ myeloid signature was assessed across myeloid populations to illustrate its distribution among distinct myeloid subsets (Figure 6G,H). To further prioritize functionally relevant signaling events, NicheNet analysis ranked high‐confidence ligands derived from CAFs subtypes (Figure 6I) and highlighted subtype‐associated ligand–receptor pairs (Figure 6J). In addition, the predicted target gene programs downstream of POSTN^+^ CAFs‐associated ligands were visualized across CAFs subtypes (Figure 6K). Together, these findings support extensive CAFs–myeloid communication in LUAD and highlight distinct subtype‐associated signaling patterns across CAFs and myeloid populations within the TME.

### 3.7. POSTN^+^ and CXCL8^+^ CAFs Signatures Associate With Adverse Clinical Outcomes and Couple With CCL3^+^ Myeloid Programs in Bulk and Spatial Settings

**Figure 7:**
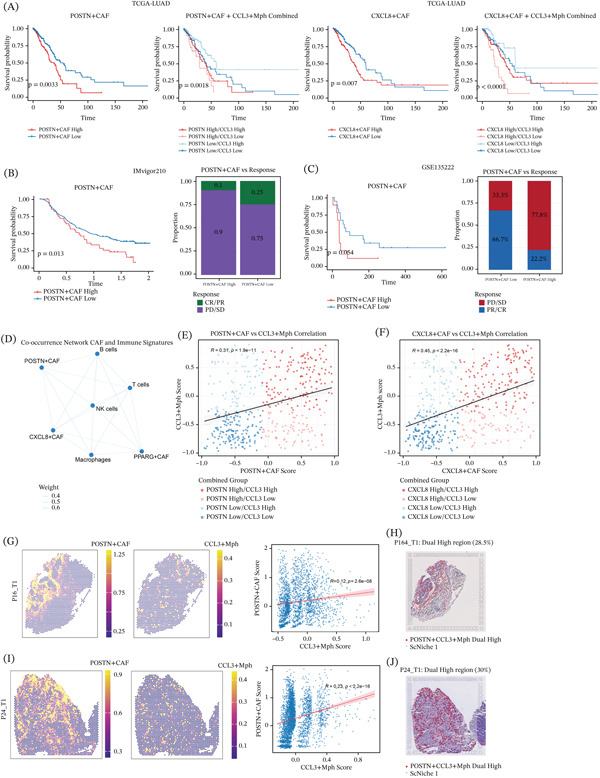
Prognostic, immunotherapeutic, and spatial characterization of CAFs subtypes in LUAD. (A) Kaplan–Meier OS curves for TCGA‐LUAD, comparing high versus low POSTN^+^CAFs/CXCL8^+^CAFs signature expression, and combined stratification with CCL3^+^Mph signature (log‐rank test). (B) OS curves and response bar plots for IMvigor210 immunotherapy cohort, stratified by POSTN^+^CAFs signature (log‐rank test). (C) OS curves and response bar plots for GSE135222 immunotherapy cohort, stratified by POSTN^+^CAFs signature (log‐rank test, Fisher′s exact test). (D) Co‐occurrence network of CAFs subtype and immune cell signatures in TCGA‐LUAD; edge thickness indicates correlation strength. (E–F) Scatter plots of (E) POSTN^+^CAFs and (F) CXCL8^+^CAFs signature scores versus CCL3^+^Mph signature scores in TCGA‐LUAD, with Pearson correlation coefficients annotated. (G, I) Spatial distribution of POSTN^+^CAFs and CCL3^+^Mph signatures in two LUAD spatial transcriptomics sections (P16_T1 and P24_T1), with correlation analyses. (H, J) scNiche analysis validation of POSTN^+^CAFs/CCL3^+^Mph dual‐high regions in serial sections of P16_T1 and P24_T1.

In the TCGA‐LUAD cohort, Kaplan–Meier analysis showed that patients with higher POSTN^+^CAFs or CXCL8^+^CAFs signature scores had worse overall survival than those with lower scores (log‐rank test; Figure 7A). Further stratification by combining CAFs subtype signatures with the CCL3^+^Mph signature separated patients into distinct risk groups, indicating that different combinations of fibroblast and myeloid programs were associated with different clinical outcomes (Figure 7A). In the immunotherapy cohorts, survival analysis and response comparison were further performed according to the POSTN^+^CAFs signature. In IMvigor210, patients with higher POSTN^+^CAFs signature scores had significantly shorter overall survival and showed a poorer response to immunotherapy than those in the low‐score group (Figure 7B). Similar analyses were also performed in GSE135222, where both survival and response status were compared between groups stratified by the POSTN^+^CAFs signature, and comparable results were observed (Figure 7C). At the bulk‐transcriptome level, co‐occurrence analysis in TCGA‐LUAD showed correlations between CAFs subtype signatures and immune cell signatures (Figure 7D). In particular, POSTN^+^CAFs and CXCL8^+^CAFs signature scores were positively correlated with the CCL3^+^Mph signature score (Figure 7E,F). Spatial transcriptomic analysis further demonstrated the distribution of POSTN^+^CAFs and CCL3^+^Mph signatures in two LUAD sections (P16_T1 and P24_T1), and correlation analysis further supported their spatial association within tissue sections. The correlation coefficients in the two samples were 0.12 and 0.23, respectively, with both *p* values < 0.001. In addition, the overlapping dual‐high regions accounted for 28.5% and 30.0% of the corresponding spatial sections, respectively (Figure 7G–J). Taken together, these findings support a close relationship between CAFs‐related and myeloid‐related programs in LUAD.

## 4. Discussion

In this study, by integrating eight independent LUAD single‐cell RNA‐seq cohorts together with bulk transcriptomic and spatial transcriptomic data, we constructed a large‐scale single‐cell atlas and systematically characterized CAFs heterogeneity, functional states, and stromal–myeloid communication patterns in LUAD [10, 15]. Several findings are particularly notable. First, LUAD contained multiple transcriptionally and functionally distinct CAFs subtypes, among which POSTN^+^ CAFs and CXCL8^+^ CAFs showed the strongest associations with tumor‐related stromal activation [13]. Second, POSTN^+^ CAFs displayed prominent enrichment of ECM remodeling, focal adhesion, PI3K–AKT, and TGF‐*β*–related programs, suggesting that this subtype may represent a fibroblast state closely linked to matrix deposition and stromal activation. Third, trajectory and regulatory analyses further indicated that CAFs heterogeneity was not purely static, but was organized along a continuous differentiation spectrum, with IKZF2^+^ CAFs located closer to the root and LHFP^+^ and POSTN^+^ CAFs occupying more terminal states. Finally, across bulk, spatial, and cell–cell communication analyses, we consistently observed a close relationship between POSTN^+^ CAFs and CCL3^+^ myeloid programs, supporting the existence of a fibroblast–myeloid interaction pattern associated with immune exclusion and adverse clinical outcome in LUAD.

Importantly, we connected these CAFs states to immune exclusion quantitatively and spatially [16]. In TCGA‐LUAD, POSTN^+^ CAFs abundance correlated with a T‐cell exclusion index, suggesting that ECM‐dominant CAFs programs align with impaired T‐cell penetration or function. Spatial transcriptomics further supported an anatomical interpretation: POSTN^+^ and CXCL8^+^ CAFs signatures localized predominantly to hypoxic, nonepithelial regions and colocalized with CD68^+^ myeloid signals. This spatial arrangement mirrors a “border” or “barrier” niche reported in multiple cancers, where fibroblast‐rich stroma and myeloid‐dominant inflammation surround tumor nests and restrict cytotoxic lymphocyte access. This limitation at the microscopic anatomical level may explain why some tumors, although immune cells can be observed in the periphery, still lack response to ICB therapy in practice, because effector cells cannot enter and attack tumor cells.

A central significance of our study is that the heterogeneity of CAFs in LUAD is not simply defined by marker genes, but has a clear, specific functional structure. Although previous single‐cell studies have suggested heterogeneity of fibroblasts in lung cancer [17, 18], our pooled analysis further shows that different CAFs subsets correspond to different pathway activity, coexpression modules, and potential regulatory statuses. Specifically, POSTN^+^ CAFs have been repeatedly associated with ECM‐related features and myofibroblast‐like features, whereas CXCL8^+^ CAFs are more likely to be associated with inflammatory signals. This distinction is important because it suggests that different CAFs states may promote tumor progression through different mechanisms. That is, CAFs cannot simply be considered stromal cell compartments; conversely, our results support the conclusion that specific CAFs states play differential roles in matrix remodeling, immunomodulation, and possibly treatment tolerance.

Among these subpopulations, POSTN^+^ CAFs are the most stable and consistent tumor‐associated population. This subpopulation is significantly enriched in tumor tissues and exhibits the highest activity in TGF‐*β* signaling, ECM–receptor interaction, and focal adhesion and is closely related to the coexpression module of CALD1, TPM4, and ITGB1. Taken together, these features suggest that POSTN^+^ CAFs are closely related to a matrix remodeling and contractile stromal phenotype. In addition, WGCNA has identified highly linked key genes such as ATP5E and GLTSCR2, suggesting that the transcriptome of subsets of CAFs may be supported by a relatively stable network structure. Although these analyses are not sufficient to demonstrate a direct causal effect, they at least suggest that specific subsets of CAFs may represent different stromal states that are critical in maintaining the LUAD fibrotic TME. This explanation is also consistent with the results of pseudo‐timing analysis that POSTN^+^ CAFs are located near terminal branches, further supporting that this subset may correspond to a more activated or differentiated fibroblast state.

Another important finding is that CAFs biology in LUAD appears to be closely linked to myeloid programs rather than acting in isolation. Cell–cell communication analysis showed extensive ligand–receptor interactions between CAFs subtypes and myeloid populations, with POSTN^+^ CAFs showing the strongest communication with one myeloid subpopulation. At the same time, bulk‐level analyses showed positive correlations between POSTN^+^ CAFs and CCL3^+^ myeloid signatures, and spatial transcriptomics further supported their local cooccurrence within the same tissue regions. Taken together, these findings support the idea that stromal and myeloid compartments may form coordinated niches in LUAD. In this context, POSTN^+^ CAFs may contribute to a structurally restrictive environment through ECM remodeling, whereas associated myeloid programs may further reinforce immune dysfunction. Our data therefore favor a model of fibroblast–myeloid cooperation rather than one in which either stromal cells or immune cells alone determine the immune landscape.

Our results also have implications for understanding immune exclusion in LUAD. The POSTN^+^ CAFs signature showed a positive correlation with a T‐cell exclusion index in TCGA‐LUAD, and POSTN^+^ as well as CXCL8^+^ CAFs programs were preferentially localized to hypoxic, nonepithelial regions in spatial transcriptomic sections. In addition, CD276 (B7‐H3) expression differed significantly between POSTN^+^ CAFs‐high and POSTN^+^ CAFs‐low groups, supporting differences in the immune checkpoint–related microenvironment according to stromal state [19]. These observations suggest that immune exclusion in LUAD may not simply reflect lack of immune infiltration, but may instead arise in the setting of a fibrotic and spatially constrained stromal niche accompanied by myeloid‐associated immune suppression. Within this framework, POSTN^+^ CAFs are particularly noteworthy because they are located at the intersection of matrix remodeling, hypoxia‐related spatial positioning, and immune‐related transcriptional changes [20].

Pseudotime and regulatory network analysis provide another layer of explanation for understanding the evolution of CAFs. The six CAFs subpopulations are not completely independent fibroblast groups but rather appear connected along a continuous differentiation trajectory. The inferred lineage topology shows that IKZF2^+^ CAFs are closer to the root state, JUND^+^ CAFs occupy an intermediate position, whereas LHFP^+^ and POSTN^+^ CAFs are situated nearer to the terminal branches. Gene modules associated with pseudotime further reveal sequential activation of ECM‐related, inflammation‐related, immune recruitment‐related, and stress response‐related programs. In this context, FOXS1 and IL24 were identified as candidate regulatory factors linked to subpopulation‐specific states. Notably, FOXS1 is preferentially enriched in POSTN^+^ CAFs, whereas IL24 shows high expression not only in POSTN^+^ CAFs but also in CXCL8^+^ CAFs, suggesting that different regulatory factors may be involved in distinct branches of CAFs activation. Although direct evidence regarding FOXS1 in lung cancer CAFs remains limited, studies in other tumor types and fibrosis‐related research have already associated FOXS1 with TGF‐*β* responsiveness, stromal activation, and epithelial–mesenchymal transition, and pan‐cancer analyses also suggest that high FOXS1 expression could be linked to a more immunosuppressive TME [21]. These observations are consistent with the speculation that FOXS1 may participate in stromal activation and fibroblast state transition in LUAD [22, 23]. In contrast, IL24 has been more extensively studied in tumor biology, especially in the context of lung cancer, where it is generally described as a tumor‐suppressive cytokine with proapoptotic and antiangiogenic effects [24]. Experimental studies in NSCLC have shown that exogenous IL24/MDA‐7 can inhibit lung cancer growth, suppress angiogenesis, and enhance sensitivity to anti‐tumor therapies [25]. However, in our data, IL24 tends to be expressed in specific CAFs‐related states and cannot be simply interpreted as a classical antitumor molecule. This expression pattern suggests that the biological role of IL24 in the TME may be context‐dependent and could also reflect stress‐related, inflammation‐related, or compensatory transcriptional programs within activated CAFs subpopulations. Overall, these findings provide a new explanatory framework for understanding how fibroblasts transition from a relatively quiescent state to terminal tumor‐associated states and indicate that FOXS1 and IL24 are candidate regulatory factors worthy of further functional validation in the future.

From the perspective of translational medicine, our results suggest that the significance of CAFs‐related features is not limited to descriptive single‐cell classification. In TCGA‐LUAD, both POSTN^+^ CAFs and CXCL8^+^ CAFs signatures were associated with poorer overall survival. Furthermore, when CAFs‐related programs were combined with CCL3^+^ myeloid programs, prognostic stratification could be further optimized, indicating that stromal–myeloid coupling may better reflect clinically significant tumor states compared with examining either the stromal or myeloid compartments alone. In external immunotherapy cohorts, higher POSTN^+^ CAFs signatures were also associated with poorer survival and less favorable treatment responses. Although these cohorts alone are not sufficient to directly demonstrate that POSTN^+^ CAFs directly mediate immunotherapy resistance, they at least support the viewpoint that this stromal program is clinically associated with a microenvironment that is less favorable for immune response. This may also have implications for future biomarker development, especially in identifying tumor patients with prominent stromal constraints and myeloid‐associated immunosuppressive features.

This study also has several limitations that need to be noted. Firstly, our conclusions are primarily based on integrated analyses of public datasets and computational inference, and thus are inherently observational. Although the consistency of results across single‐cell, bulk, spatial transcriptomics, and immunotherapy cohorts increases our confidence in the robustness of the conclusions, establishing whether the proposed fibroblast–myeloid cell interactions are mechanistically necessary for immune exclusion still requires direct experimental validation. Future studies could address this question further using in vitro coculture systems, perturbation experiments targeting candidate ligand–receptor axes, and in vivo models to assess whether disrupting specific fibroblast–myeloid interactions can relieve stromal constraints and enhance antitumor immunity. Secondly, current spatial analyses support local coenrichment and spatial correlation, but cannot yet provide definitive evidence of direct physical contact or functional dependence between POSTN^+^ CAFs and CCL3^+^ myeloid cells. Future work could combine multiplex immunofluorescence, immunohistochemistry, and higher resolution spatial omics technologies to further validate their spatial proximity and tissue architecture in LUAD tissues. Third, the inferred trajectory structure should be interpreted as a model of transcriptional progression rather than a direct reconstruction of true biological lineage history. Future studies integrating lineage tracing, time‐resolved perturbation, or longitudinal sampling strategies would help clarify the developmental relationships among CAFs subsets. Finally, the immunotherapy validation, although supportive, is still limited by the availability and size of public cohorts, and additional LUAD‐ or NSCLC‐specific datasets would further strengthen the translational interpretation. In the future, prospective validation in larger immunotherapy‐treated LUAD cohorts, together with integrated analyses of stromal, myeloid, and clinical response features, will be important to determine whether the identified CAFs‐associated programs may serve as robust biomarkers or potential therapeutic targets.

In summary, our study provides a multilevel view of CAFs heterogeneity in LUAD and demonstrates that different CAFs subtypes are associated with distinct stromal, immune, and myeloid‐related programs. By integrating coexpression analysis, trajectory inference, regulatory analysis, cell–cell communication, and spatial validation, our results suggest that fibroblast–myeloid coupling is an important feature of the LUAD microenvironment. Overall, these findings deepen our understanding of CAFs biology in LUAD and offer a useful framework for future studies aimed at targeting stromal barriers and improving antitumor immune responses.

## Funding

This work was supported by the National Natural Science Foundation of China (Grant No. 82101869); the Natural Science Foundation for Young Scientists of Hunan Province (Grant Nos. 2021JJ40471 and 2021JJ40478); the Scientific Research Project of Hunan Education Department (Grant No. 21B0404); and the Hunan University Students Innovation and Entrepreneurship Training Program (Grant Nos. S202410555050, S202410555117, S202410555243 and S202510555311).

## Conflicts of Interest

The authors declare no conflicts of interest.

## Supporting information


**Supporting Information** Additional supporting information can be found online in the Supporting Information section. Figure S1 is a figure for better understanding the functional enrichment analyses of different WGCNA modules. It also shows the integration of seven independent single‐cell RNA sequencing (scRNA‐seq) datasets.

## Data Availability

The data that support the findings of this study are openly available in GSE148071, GSE131907, GSE179994, GSE127465, and GSE207422 at https://www.ncbi.nlm.nih.gov/geo/query.

## References

[1] Riely G. J. , Wood D. E. , Aisner D. L. , Axtell A. L. , Bauman J. R. , Bharat A. , Chang J. Y. , Desai A. , Dilling T. J. , Dowell J. , Durm G. A. , Gettinger S. , Grotz T. E. , Gubens M. A. , Juloori A. , Lackner R. P. , Lanuti M. , Levy B. , Lin J. , Loo BW Jr , Lovly C. M. , Maldonado F. , Morgensztern D. , Mullikin T. C. , Ng T. , Owen D. , Owen D. H. , Patil T. , Polanco P. M. , Riess J. , Mendez A. L. R. , Shapiro T. A. , Singh A. P. , Stevenson J. , Tam A. , Tanvetyanon T. , Yanagawa J. , Yau E. , Yun K. , Gregory K. , and Hang L. , Non-Small Cell Lung Cancer, Version 4.2026, NCCN Clinical Practice Guidelines in Oncology, Journal of the National Comprehensive Cancer Network. (2026) 24, no. 4, e260017, 10.6004/jnccn.2026.0017, 41956107. 41956107

[2] Baudin E. , Durand A. , Buikhuisen W. , Capdevila J. , Caplin M. , Deroose C. M. , Dromain C. , Faggiano A. , Filosso P. L. , Kaltsas G. , Volante M. , Walter T. , and Garcia-Carbonero R. , European Society of Neuroendocrine Tumors (ENETS) 2025 Guidance Paper for Lung and Thymic Carcinoids, Journal of Neuroendocrinology. (2026) 38, no. 4, e70174, 10.1111/jne.70174, 41941890. 41941890 PMC13053111

[3] Joyce J. A. and Fearon D. T. , T Cell Exclusion, Immune Privilege, and the Tumor Microenvironment, Science. (2015) 348, no. 6230, 74–80, 10.1126/science.aaa6204, 2-s2.0-84928896256, 25838376. 25838376

[4] Chen D. S. and Mellman I. , Elements of Cancer Immunity and the Cancer-Immune Set Point, Nature. (2017) 541, no. 7637, 321–330, 10.1038/nature21349, 2-s2.0-85016548232, 28102259. 28102259

[5] Mitsuhashi A. , Koyama K. , Ogino H. , Matsuo R. , Thi Nguyen N. , Yabuki Y. , Ozaki R. , Tsukazaki Y. , Morita Y. , Yoshida A. , Murakami K. , Sato S. , Kawano H. , Nokihara H. , Inagaki Y. , Shinohara T. , Hanibuchi M. , Takizawa H. , and Nishioka Y. , Clock Pathway Inhibitor Overcomes Tumor Immune-Exclusion via Regulation of Fibrocyte Differentiation, NPJ Precision Oncology. (2025) 9, no. 1, 10.1038/s41698-025-01066-6, 40764393. PMC12326006 40764393

[6] Biffi G. and Tuveson D. A. , Diversity and Biology of Cancer-Associated Fibroblasts, Physiological Reviews. (2021) 101, no. 1, 147–176, 10.1152/physrev.00048.2019, 32466724. 32466724 PMC7864232

[7] Sahai E. , Astsaturov I. , Cukierman E. , DeNardo D. , Egeblad M. , Evans R. M. , Fearon D. , Greten F. R. , Hingorani S. R. , Hunter T. , Hynes R. O. , Jain R. K. , Janowitz T. , Jorgensen C. , Kimmelman A. C. , Kolonin M. G. , Maki R. G. , Powers R. S. , Puré E. , Ramirez D. C. , Scherz-Shouval R. , Sherman M. H. , Stewart S. , Tlsty T. D. , Tuveson D. A. , Watt F. M. , Weaver V. , Weeraratna A. T. , and Werb Z. , A Framework for Advancing Our Understanding of Cancer-Associated Fibroblasts, Nature Reviews Cancer. (2020) 20, no. 3, 174–186, 10.1038/s41568-019-0238-1, 31980749. 31980749 PMC7046529

[8] Forsthuber A. , Aschenbrenner B. , Korosec A. , Jacob T. , Annusver K. , Krajic N. , Kholodniuk D. , Frech S. , Zhu S. , Purkhauser K. , Lipp K. , Werner F. , Nguyen V. , Griss J. , Bauer W. , Soler Cardona A. , Weber B. , Weninger W. , Gesslbauer B. , Staud C. , Nedomansky J. , Radtke C. , Wagner S. N. , Petzelbauer P. , Kasper M. , and Lichtenberger B. M. , Cancer-Associated Fibroblast Subtypes Modulate the Tumor-Immune Microenvironment and Are Associated With Skin Cancer Malignancy, Nature Communications. (2024) 15, no. 1, 10.1038/s41467-024-53908-9, 39516494. PMC11549091 39516494

[9] Browaeys R. , Saelens W. , and Saeys Y. , NicheNet: Modeling Intercellular Communication by Linking Ligands to Target Genes, Nature Methods. (2020) 17, no. 2, 159–162, 10.1038/s41592-019-0667-5, 31819264. 31819264

[10] Yang C. , Liu W. , Powell C. A. , and Wang Q. , Heterogeneity and Therapeutic Implications of Cancer-Associated Fibroblasts in Lung Cancer: Recent Advances and Future Perspectives, Chinese Medical Journal Pulmonary and Critical Care Medicine. (2024) 2, no. 4, 240–249, 10.1016/j.pccm.2024.08.009, 39834587. 39834587 PMC11742357

[11] Chen C. , Guo Q. , Liu Y. , Hou Q. , Liao M. , Guo Y. , Zang Y. , Wang F. , Liu H. , Luan X. , Liang Y. , Guan Z. , Li Y. , Liu H. , Dong X. , Zhang X. , Liu J. , and Xu Q. , Single-Cell and Spatial Transcriptomics Reveal POSTN+ Cancer-Associated Fibroblasts Correlated With Immune Suppression and Tumour Progression in Non-Small Cell Lung Cancer, Clinical and Translational Medicine. (2023) 13, no. 12, e1515, 10.1002/ctm2.1515, 38115703. 38115703 PMC10731139

[12] Hirano Y. , Suzuki H. , Nakayama J. , Yamamoto T. , Hirata E. , Araya J. , Fujita Y. , and Yamamoto Y. , An Integrated Single-Cell Lung Cancer Atlas Reveals Distinct Fibroblast Phenotypes Between Adenocarcinoma and Squamous Cell Carcinomas, NPJ Precision Oncology. (2026) 10, no. 1, 10.1038/s41698-026-01279-3, 41577803. PMC12920623 41577803

[13] Pietrobon V. and Marincola F. M. , Hypoxia and the Phenomenon of Immune Exclusion, Journal of Translational Medicine. (2021) 19, no. 1, 9, 10.1186/s12967-020-02667-4, 33407613. 33407613 PMC7788724

[14] Piwocka O. , Piotrowski I. , Suchorska W. M. , and Kulcenty K. , Dynamic Interactions in the Tumor Niche: How the Cross-Talk Between CAFs and the Tumor Microenvironment Impacts Resistance to Therapy, Frontiers in Molecular Biosciences. (2024) 11, 1343523, 10.3389/fmolb.2024.1343523. 38455762 PMC10918473

[15] Wong K. Y. , Cheung A. H. K. , Chen B. , Chan W. N. , Yu J. , Lo K. W. , Kang W. , and To K. F. , Cancer-Associated Fibroblasts in Nonsmall Cell Lung Cancer: From Molecular Mechanisms to Clinical Implications, International Journal of Cancer. (2022) 151, no. 8, 1195–1215, 10.1002/ijc.34127, 35603909. 35603909 PMC9545594

[16] Peyraud F. , Guégan J. P. , Rey C. , Lara O. , Odin O. , del Castillo M. , Vanhersecke L. , Coindre J. M. , Clot E. , Brunet M. , Grellety T. , Tasseel A. , Moulec S. L. , Johnston R. J. , Bessede A. , and Italiano A. , Spatially Resolved Transcriptomics Reveal the Determinants of Primary Resistance to Immunotherapy in NSCLC With Mature Tertiary Lymphoid Structures, Cell Reports Medicine. (2025) 6, no. 2, 101934, 10.1016/j.xcrm.2025.101934, 39909044. 39909044 PMC11866545

[17] Kim D. , Kim J. S. , Cheon I. , Kim S. R. , Chun S. H. , Kim J. J. , Lee S. , Yoon J. S. , Hong S. A. , Won H. S. , Kang K. , Ahn Y. H. , and Ko Y. H. , Identification and Characterization of Cancer-Associated Fibroblast Subpopulations in Lung Adenocarcinoma, Cancers. (2022) 14, no. 14, 10.3390/cancers14143486, 35884546. PMC9324153 35884546

[18] Mathieson L. , Koppensteiner L. , Dorward D. A. , O′Connor R. A. , and Akram A. R. , Cancer-Associated Fibroblasts Expressing Fibroblast Activation Protein and Podoplanin in Non-Small Cell Lung Cancer Predict Poor Clinical Outcome, British Journal of Cancer. (2024) 130, no. 11, 1758–1769, 10.1038/s41416-024-02671-1, 38582812. 38582812 PMC11130154

[19] Omran K. , Kousar I. , Lu J. , and Tan M. , B7-H3 (CD276) in Exosome Biogenesis and the Tumor Microenvironment: A New Therapeutic Nexus, Cell Communication and Signaling. (2026) 10.1186/s12964-026-02872-6, 41964005. 41964005

[20] Lu Y. , Wu Y. , Xu X. , Feng Y. , and Wang N. , Cancer-Associated Fibroblasts: Central Players in Cancer Hallmarks and Therapeutic Resistance, Cell Communication and Signaling. (2025) 23, no. 1, 534, 10.1186/s12964-025-02516-1, 41444607. 41444607 PMC12729356

[21] Melchionna R. , Trono P. , Di Carlo A. , Di Modugno F. , and Nisticò P. , Transcription Factors in Fibroblast Plasticity and CAF Heterogeneity, Journal of Experimental & Clinical Cancer Research. (2023) 42, no. 1, 10.1186/s13046-023-02934-4, 38124183. PMC10731891 38124183

[22] Bates E. A. , Kipp Z. A. , Lee W. H. , Martinez G. J. , Weaver L. , Becker K. N. , Pauss S. N. , Creeden J. F. , Anspach G. B. , Helsley R. N. , Xu M. , Bruno M. E. C. , Starr M. E. , and Hinds T. D. , FOXS1 Is Increased in Liver Fibrosis and Regulates TGF*β* Responsiveness and Proliferation Pathways in Human Hepatic Stellate Cells, Journal of Biological Chemistry. (2024) 300, no. 3, 105691, 10.1016/j.jbc.2024.105691, 38280429. 38280429 PMC10878791

[23] Liu Y. , Tu M. , and Wang L. , Pan-Cancer Analysis Predicts FOXS1 as a Key Target in Prognosis and Tumor Immunotherapy, International Journal of General Medicine. (2022) Volume 15, 2171–2185, 10.2147/IJGM.S354195, 35241932. 35241932 PMC8887970

[24] Panneerselvam J. , Srivastava A. , Mehta M. , Chen A. , Zhao Y. D. , Munshi A. , and Ramesh R. , IL-24 Inhibits Lung Cancer Growth by Suppressing GLI1 and Inducing DNA Damage, Cancers. (2019) 11, no. 12, 10.3390/cancers11121879, 31783569. PMC6966580 31783569

[25] Xie Y. , Sheng W. , Xiang J. , Ye Z. , Zhu Y. , Chen X. , and Yang J. , Recombinant Human IL-24 Suppresses Lung Carcinoma Cell Growth Via Induction of Cell Apoptosis and Inhibition of Tumor Angiogenesis, Cancer Biotherapy and Radiopharmaceuticals. (2008) 23, no. 3, 310–320, 10.1089/cbr.2007.0453, 2-s2.0-46449123597, 18593364. 18593364

