# Supplementary material for: A Spatially Constrained Fibroblast–Myeloid Program Associates With Immune Exclusion and Poor Prognosis in Lung Adenocarcinoma
Source: Hum Mutat. 2026 May 26;2026:8218916. doi: 10.1155/humu/8218916 (PMC13202445; doi:10.1155/humu/8218916)
Supplement: Supplementary file 1 — Supporting Information Additional supporting information can be found online in the Supporting Information section. Figure S1 is a figure for better understanding the functional enrichment analyses of different WGCNA modules. It also shows the integration of seven independent single‐cell RNA sequencing (scRNA‐seq) datasets. [file HUMU-2026-8218916-s001.pdf]

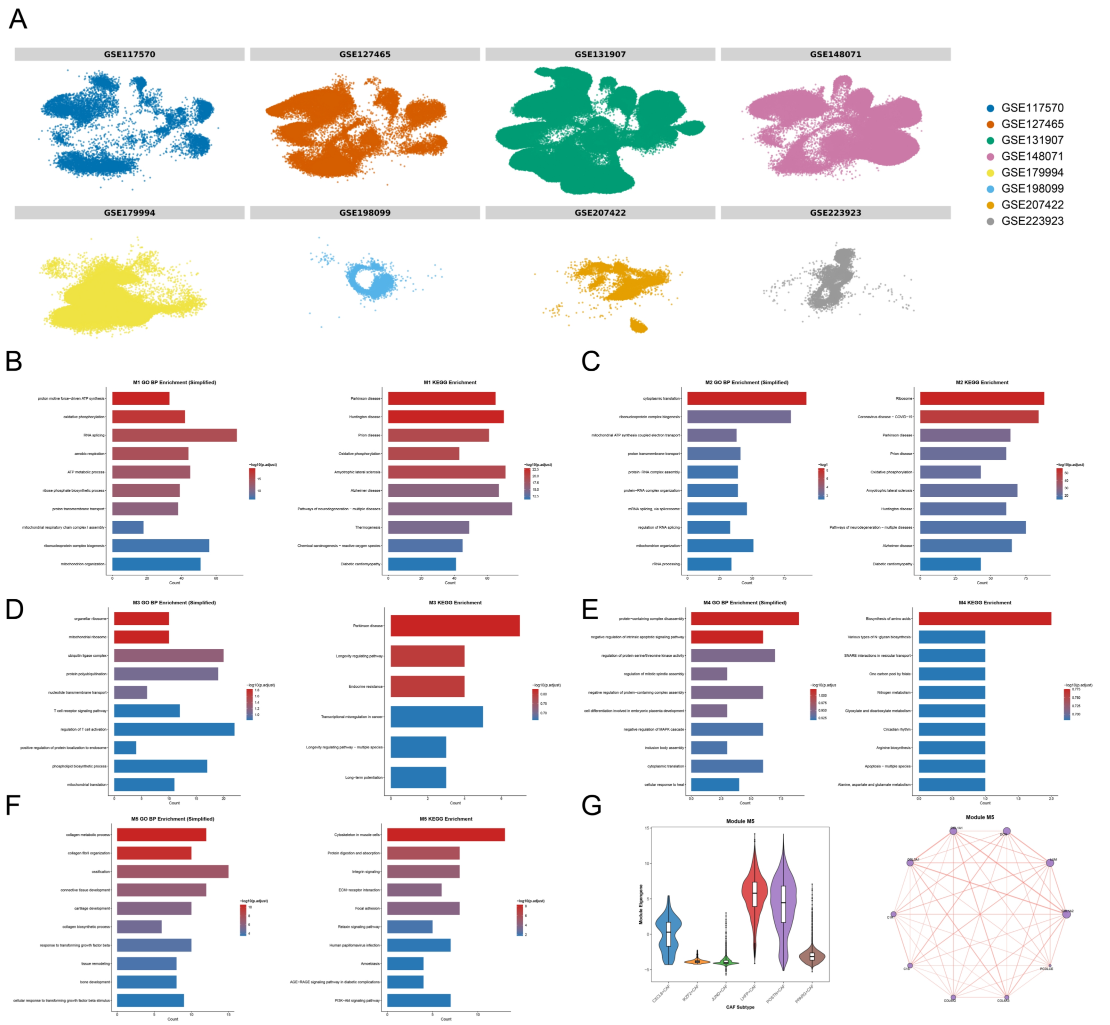

Supplementary Figure 1. Characterization of Single-Cell Datasets and Functional Enrichment Analyses of WGCNA Modules

(A) Uniform Manifold Approximation and Projection (UMAP) visualization of the integration of seven independent single-cell RNA sequencing (scRNA-seq) datasets (GSE148071, GSE131907, GSE179994, GSE127465, GSE207422, GSE198099, GSE117570, GSE223923). Each color represents a distinct dataset batch, indicating successful integration and minimal batch effect.

(B–F) Gene Ontology (GO) and Kyoto Encyclopedia of Genes and Genomes (KEGG) pathway enrichment analyses of the five weighted gene co-expression network analysis (WGCNA) modules (M1–M5). Bar plots display the top enriched terms in biological process (BP), cellular component (CC), molecular function (MF), and KEGG pathways. The color gradient represents the significance level of enrichment (−log10(P-value)), while the bar length indicates the gene ratio.

(G)Co-expression network visualization for modules M5(yellow). Nodes represent hub genes; edges represent significant co-expression (correlation strength > 0.8).
